# Bayesian inference of biochemical kinetic parameters using the linear noise approximation

**DOI:** 10.1186/1471-2105-10-343

**Published:** 2009-10-19

**Authors:** Michał Komorowski, Bärbel Finkenstädt, Claire V Harper, David A Rand

**Affiliations:** 1 Department of Statistics, University of Warwick, Coventry, UK; 2 Systems Biology Centre, University of Warwick, Coventry, UK; 3 Mathematics Institute, University of Warwick, Coventry, UK; 4 Department of Biology, University of Liverpool, Liverpool, UK

## Abstract

**Background:**

Fluorescent and luminescent gene reporters allow us to dynamically quantify changes in molecular species concentration over time on the single cell level. The mathematical modeling of their interaction through multivariate dynamical models requires the deveopment of effective statistical methods to calibrate such models against available data. Given the prevalence of stochasticity and noise in biochemical systems inference for stochastic models is of special interest. In this paper we present a simple and computationally efficient algorithm for the estimation of biochemical kinetic parameters from gene reporter data.

**Results:**

We use the linear noise approximation to model biochemical reactions through a stochastic dynamic model which essentially approximates a diffusion model by an ordinary differential equation model with an appropriately defined noise process. An explicit formula for the likelihood function can be derived allowing for computationally efficient parameter estimation. The proposed algorithm is embedded in a Bayesian framework and inference is performed using Markov chain Monte Carlo.

**Conclusion:**

The major advantage of the method is that in contrast to the more established diffusion approximation based methods the computationally costly methods of data augmentation are not necessary. Our approach also allows for unobserved variables and measurement error. The application of the method to both simulated and experimental data shows that the proposed methodology provides a useful alternative to diffusion approximation based methods.

## Background

The estimation of parameters in biokinetic models from experimental data is an important problem in Systems Biology. In general the aim is to calibrate the model so as to reproduce experimental results in the best possible way. The solution of this task plays a key role in interpreting experimental data in the context of dynamic mathematical models and hence in understanding the dynamics and control of complex intracellular chemical networks and the construction of synthetic regulatory circuits [1]. Among biochemical kinetic systems, the dynamics of gene expression and of gene regulatory networks are of particular interest. Recent developments of fluorescent microscopy allow us to quantify changes in protein concentration over time in single cells (e.g. [2,3]) even with single molecule precision (see [4] for review). Therefore an abundance of data is becoming available to estimate parameters of mathematical models in many important cellular systems.

Single cell imaging techniques have revealed the stochastic nature of biochemical reactions (see [5] for review) that most often occur far from thermodynamic equilibrium [6] and may involve small copy numbers of reacting macromolecules [7]. This inherent stochasticity implies that the dynamic behaviour of one cell is not exactly reproducible and that there exists stochastic heterogeneity between cells. The disparate biological systems, experimental designs and data types impose conditions on the statistical methods that should be used for inference [8-10]. From the modeling point of view the current common consensus is that the most exact stochastic description of the biochemical kinetic system is provided by the chemical master equation (CME) [11]. Unfortunately, for many tasks such as inference the CME is not a convenient mathematical tool and hence various types of approximations have been developed. The three most commonly used approximations are [12]:

1. The macroscopic rate equation (MRE) approach which describes the thermodynamic limit of the system with ordinary differential equations and does not take into account random fluctuations due to the stochasticity of the reactions.

2. The diffusion approximation (DA) which provides stochastic differential equation (SDE) models where the stochastic perturbation is introduced by a state dependant Gaussian noise.

3. The linear noise approximation (LNA) which can be seen as a combination because it incorporates the deterministic MRE as a model of the macroscopic system and the SDEs to approximatively describe the fluctuations around the deterministic state.

Statistical methods based on the MRE have been most widely studied [8,13-15]. They require data based on large populations. The main advantages of this method are its conceptual simplicity and the existence of extensive theory for differential equations. However, single cells experiments and studies of noise in small regulatory networks created the need for statistical tools that are capable to extract information from fluctuations in molecular species. Few methods used CME to address this. Algorithm, proposed by [16], approximated the likelihood function, the other, suggested by [17] simulated it using Monte Carlo methods. Recently, also a method based on the exact likelihood [18] has been developed. Although, substantial progress has been made in numerical methods for solving CME, inference algorithms based on the CME are computationally intensive and difficult to apply to problems of realistic size and complexity [19]. Another group of methods is based on the DA [9,20]. This uses likelihood approximation methods (e.g. [21]) that are computationally intensive and require sampling from high dimensional posterior distributions. Inference about the volatility process becomes difficult for low frequency data that are not directly measured at the molecular level [10,20]. The aim of this study is to investigate the use of the LNA as a method for inference about kinetic parameters of stochastic biochemical systems. We find that the LNA approximation provides an explicit Gaussian likelihood for models with hidden variables and measurement error and is therefore simpler to use and computationally efficient. To account for prior information on parameters our methodology is embedded in the Bayesian paradigm. The paper is structured as follows: We first provide a description of the LNA based modeling approach and then formulate the relevant statistical framework. We then study its applicability in four examples, based on both simulated and experimental data, that clarify principles of the method. Additional file 1 contains details of mathematical and statistical modeling, particularly comparison of the proposed method with an algorithm based on the DA.

## Methods

The chemical master equation (CME) is the primary tool to model the stochastic behaviour of a reacting chemical system. It describes the evolution of the joint probability distribution of the number of different molecular species in a spatially homogeneous, well stirred and thermally equilibrated chemical system [11].

Even though these assumptions are not necessarily satisfied in living organisms the CME is commonly regarded as the most realistic model of biochemical reactions inside living cells. Consider a general system of *N *chemical species inside a volume Ω and let **X **= (*X*_1_,..., *X*_*N*_)^*T *^denote the number and **x **= **X**/Ω the concentrations of molecules. The stoichiometry matrix **S **= {*S*_*ij*_}_*i *= 1,2...*N*; *j *= 1,2...*R *_describes changes in the population sizes due to *R *different chemical events, where each *S*_*ij *_describes the change in the number of molecules of type *i *from *X*_*i *_to *X*_*i *_+ *S*_*ij *_caused by an event of type *j*. The probability that an event of type *j *occurs in the time interval [*t*, *t *+ *dt*) equals (**x**, Ω, *t*)Ω*dt*. The functions (**x**, Ω, *t*) are called *mesoscopic transition rates*. This specification leads to a Poisson birth and death process where the probability *h*(**X**, *t*) that the system is in the state **X **at time *t *is described by the CME [12] which is given in Additional file 1. It is straightforward to verify that the first order terms of a Taylor expansian of the CME in powers of  are given by the following MRE

(1)

where *ϕ*_*i *_= lim_Ω→∞, *X*→∞ _*X*_*i*_/Ω, *φ *= (*ϕ*_1_,..., *ϕ*_*N*_)^*T *^and .

Including also the second order terms of this expansion produces the LNA

(2)

which decomposes the state of the system into a deterministic part *φ *as solution of the MRE in (1) and a stochastic process *ξ *described by an Itô diffusion equation

(3)

where *W*(*t*) denotes *R *dimensional Wiener process,  and *f*_*i *_= *f*_*i*_(*φ*) (see Additional file 1 for derivation).

The rationale behind the expansion in terms of  is that for constant average concentrations relative fluctuations will decrease with the inverse of the square root of volume [22]. Therefore the LNA is accurate when fluctuations are sufficiently small in relation to the mean (large Ω). Hence, the natural measure of adequacy of the LNA is the coefficient of variation i.e. ratio of the standard deviation to the mean (see Additional file 1). Validity of this approximation is also discussed in details in [22,23]. In addition it can be shown that the process describing the deviation from the deterministic state  converges weakly to the diffusion (3) as Ω → ∞ [24]. In order to use the LNA in a likelihood based inference method we need to derive transition densities of the process **x**.

### Transition densities

The LNA provides solutions that are numerically or analytically tractable because the MRE in (1) can be solved numerically and the linear SDE in (3) for an initial condition *ξ*(*t*_*i*_) =  has a solution of the form [25]

(4)

where the integral is in the Itô sense and (*s*) is the fundamental matrix of the non-autonomous system of ODEs

(5)

The Itô integral of a deterministic function is a Gaussian random variable [26], therefore equations (4), (5) imply that the transition densities of the process *ξ *are Gaussian [26] (throughout the paper we use 'Gaussian' or 'normal' shortly to denote either a univariate or a multivariate normal distribution depending on the context)

(6)

where Θ denotes a vector of all model parameters, *ψ*(·|*μ*_*i*-1_, Ξ_*i*-1_) is the normal density with mean *μ*_*i*-1 _and covariance matrix Ξ_*i*-1 _specified by

(7)

It follows from (2) and (6) that the transition densities of **x **are normal

(8)

The properties of the normal distribution allow us to construct a convenient inference framework that is reminiscent of the Kalman filtering methodology (see e.g. [27]).

### Inference

It is rarely possible to observe the time evolution of all molecular components participating in the system of interest [28]. Therefore, we partition the process **x**_*t *_into those components **y**_*t *_that are observed and those **z**_*t *_that are unobserved.

Let ,  and  denote the time-series that comprise the values of processes **x**, **y **and **z**, respectively, at times *t*_0_,..., *t*_*n*_. Here and throughout the paper we use the same letter to denote the stochastic process and its realization.

Our aim is to estimate the vector of unknown parameters Θ from a sequence of measurements . The initial condition *φ*(*t*_0_) is parameterized as an element of Θ. Given the Markov property of the process **x **the augmented likelihood *P*(, |Θ) is given by

(9)

where  are Gaussian densities specified in (8), and  is an initial density assumed to be normal for mathematical convenience. It can then be shown that (see Additional file 1)  is Gaussian. Therefore

(10)

where *φ*(·|*φ*(*t*_0_),..., *φ*(*t*_*n*_), ) is Gaussian density with mean vector (*φ*(*t*_0_),..., *φ*(*t*_*n*_)) and covariance matrix  whose elements can be calculated numerically in a straightforward way (see Additional file 1). Since the marginal distributions are also Gaussian it follows that the likelihood function *P*(|Θ) can be obtained from the augmented likelihood (10)

(11)

where the covariance matrix Σ = {Σ^(*i*, *j*)^}_*i*, *j *= 0,..., *n *_is a sub-matrix of  such that  and *φ*_*y *_is the vector consisting of the observed components of *φ*.

Fluorescent reporter data are usually assumed to be proportional to the number of fluorescent molecules [29] and measurements are subject to *measurement error*, i.e. errors that do not influence the stochastic dynamics of the system. We therefore assume that instead of the matrix  our data have the form . The parameter *λ *is a proportionality constant (it is straightforward to generalize for the case with different proportionality constants for different molecular components) and  denotes a random vector for additive measurement error. For mathematical convenience we assume that the joint distribution of the measurement error is normal with mean 0 and known covariance matrix Σ_ϵ_, i.e. . If measurement errors are independent with a constant variance  then . Equation (11) implies that the likelihood function can be written as

(12)

Since for given data  the likelihood function (12) can be numerically evaluated, any likelihood based inference is straightforward to implement. Using Bayes' theorem, the posterior distribution *P*(Θ|) satisfies the relation [30]

(13)

We use the standard Metropolis-Hastings (MH) algorithm [30] to sample from the posterior distribution in (13).

## Results and Discussion

In order to study the use of the LNA method for inference we have selected four examples which are related to commonly used quantitative experimental techniques such as measurements based on reporter gene constructs and reporter assays based on Polymerase Chain Reaction (e.g. RT-PCR, Q-PCR). For expository reasons, all case studies consider a model of single gene expression.

### Model of single gene expression

Although gene expression involves various biochemical reactions it is essentially modeled in terms of only three biochemical species (DNA, mRNA, protein) and four reaction channels (transcription, mRNA degradation, translation, protein degradation) [31-33]. The stoichiometry matrix has the form

(14)

where rows correspond to molecular species and columns to reaction channels. Let **x **= (*r, p*) denote concentrations of mRNA and protein, respectively. For the reaction rates

(15)

we can derive the following macroscopic rate equations

(16)

For the general case it is assumed that the transcription rate *k*_*R*_(*t*) is time-dependent, reflecting changes in the regulatory environment of the gene such as the availability of transcription factors or chromatin structure.

Using (14), (15) and (16) in (3) we obtain the following SDEs describing the deviation from the macroscopic state (see section 3.1.4 of Additional file 1 for derivation)

(17)

We will refer to the model in (16) and (17) as the *simple model *of single gene expression.

In order to test our method on a nonlinear system we will also consider the case of an autoregulated network where the transcription rate of the gene is a function of the concentration of the protein that the gene codes for and where the protein is a transcription factor that inhibits the production of its own mRNA. This is parameterized by a Hill function [31] where *k*_*R*_(*t*) now describes the maximum rate of transcription, *H *is a dissociation constant and *n*_*H *_is a Hill coefficient.

Thus, the nonlinear autoregulatory model the system is described by the MRE

(18)

and the SDEs

(19)

where . We refer to this model as the *autoregulatory model *of single gene expression. The two models constitute the basis of our inference studies below.

### Inference from fluorescent reporter gene data for the simple model of single gene expression

To test the algorithm we first use the simple model of single gene expression. We generate data according to the stoichiometry matrix (14) and rates (15) using Stochastic Simulation Algorithm (SSA) [34] and sample it at discrete time points. We then generate artificial data that are proportional to the simulated protein data with added normally distributed measurement error with known variance . Furthermore we assume that mRNA levels are unobserved. The volume of the system Ω is unknown and we put Ω = 1 so that concentration equals the number of molecules. Thus the data are of the form

(20)

where  is the simulated protein concentration, *λ *is an unknown proportionality constant and  is measurement error. For the purpose of our example we model the transcription function by

(21)

This form of transcription corresponds to an experiment, where transcription increases for *t *≤ *b*_3 _as a result of being induced by an environmental stimulus and for *t *> *b*_3 _decreases towards a baseline level *b*_4_.

We assume that at time *t*_0 _(*t*_0 _<<*b*_3_) the system is in a stationary state. Therefore, the initial condition of the MRE is a function of unknown parameters (*ϕ*_*R*_(*t*_0_), *ϕ*_*P*_(*t*_0_)) = (*b*_4_/*γ*_*R*_, *b*_4_*k*_*P*_/*γ*_*R*_*γ*_*P*_).

To ensure identifiability of all model parameters we assume that informative prior distributions for both degradation rates are available. Priors for all other parameters were specified to be non-informative. To infer the vector of unknown parameters



we sample from the posterior distribution



using the standard MH algorithm. The distribution P(|Θ) is given by (12).

**Figure 1:**
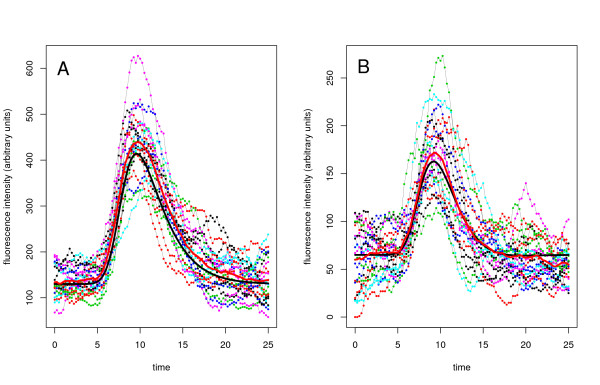
**Protein timeseries generated using Gillespie's algorithm for the simple A and autoregulatory B models of single gene expression with added measurement error **( = 9). Initial conditions for mRNA and protein were sampled from Poisson distributions with means equal to the stationary means of the system with equal constant transcription rate *b*_4_. In the autoregulatory case we set *H *= *b*_4_*k*_*P*_/2*γ*_*R*_*γ*_*P*_. In each panel 20 time series are presented. The deterministic and average trajectories are plotted in bold black and red lines respectively. Corresponding mRNA trajectories (not used for inference) are presented in Additional file 1.

**Figure 2:**
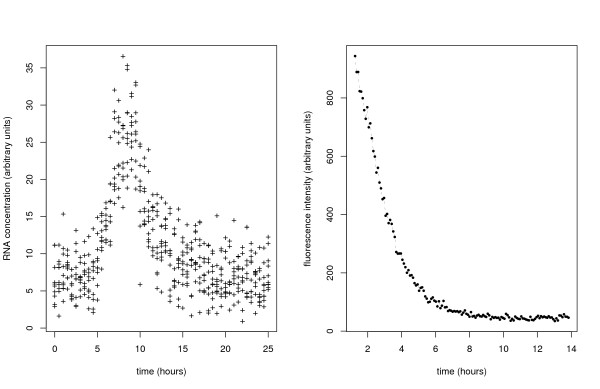
**Left: PCR based reporter assay data simulated with Gillespie's algorithm using parameters presented in Table 2 and extracted 51 times (n = 50), every 30 minutes with an independently and normally distributed error **( = 9). Each cross correspond to the end of simulated trajectory, so that the data drawn are of form (22). Since number of RNA molecules is know upto proportionality constant y-axis is in arbitrary units. Time on x-axis is expressed in hours. Estimates inferred form this data are shown in column *Estimate 1 *in Table 2. **Right**: Fluorescence level from cycloheximide experiment is plotted against time (in hours). Subsequent dots correspond to measurements taken every 6 minutes.

The protein level of the simulated trajectory is sampled every 15 minutes and a sample size of 101 points obtained. We perform inference for two simulated data sets: estimate 1 is based on a single trajectory while estimate 2 represents a larger data set using 20 sampled trajectories (see Figure 1A). All prior specifications, parameters used for the simulations and inference results are presented in Table 1A. Estimate 1 demonstrates that it is possible to infer all parameters from a single, short length time series with a realistically achievable time resolution. Estimate 2 shows that usage of the LNA does not seem to result in any significant bias. A bias has not been detected despite the very small number of mRNA molecules (5 to 35 - Figure 2A in Additional file 1) and protein molecules (100 to 500 - Figure 1A). The coefficient of variation varied between approximately 0.15 and 0.4 for both molecular species (Figure 1 in the Additional file 1).

**Table 1:** Inference results for (A) the simple model and (B) autoregulatory model of single gene expression

**(A)** **Param**.	**Prior**	**Value**	**Estimate 1**	**Estimate 2**
*γ*_*R*_	Γ (0.44,10^-2^)	0.44	0.43 (0.27-0.60)	0.49 (0.38-0.61)
*γ*_*P*_	Γ (0.52,10^-2^)	0.52	0.51 (0.35-0.67)	0.49 (0.38-0.61)
*k*_*P*_	Exp(100)	10.00	21.09 (3.91-67.17)	11.41 (7.64-16.00)
*λ*	Exp(100)	1.00	1.42 (0.60-2.57)	1.08 (0.76-1.36)
*b*_0_	Exp(100)	15.00	6.80 (0.97-18.43)	12.78 (9.80-15.33)
*b*_1_	Exp(1)	0.40	0.79 (0.05-3.02)	0.29 (0.18-0.43)
*b*_2_	Exp(1)	0.40	0.77 (0.08-2.79)	0.77 (0.32-1.59)
*b*_3_	Exp(10)	7.00	6.13 (4.41-7.85)	7.25 (6.79-7.55)
*b*_4_	Exp(100)	3.00	0.94 (0.11-2.88)	2.87 (2.11-3.52)

**(B)** **Param**.	**Prior**	**Value**	**Estimate 1**	**Estimate 2**

*γ*_*R*_	Γ (0.44,10^-2^)	0.44	0.44 (0.27-0.60)	0.42 (0.32-0.54)
*γ*_*P*_	Γ (0.52,10^-2^)	0.52	0.49 (0.33-0.65)	0.49 (0.36-0.61)
*k*_*P*_	Exp(100)	10.00	14.86 (3.18-47.97)	9.35 (5.87-13.15)
*λ*	Exp(100)	1.00	1.26 (0.48-2.30)	1.15 (0.81-1.50)
*b*_0_	Exp(100)	15.00	5.99 (0.20-23.06)	13.47 (9.24-17.13)
*b*_1_	Exp(1)	0.40	0.59 (0.01-2.75)	0.27 (0.14-0.53)
*b*_2_	Exp(1)	0.40	0.92 (0.05-2.92)	0.83 (0.21-3.52)
*b*_3_	Exp(10)	7.00	6.53(0.74-14.69)	7.27 (6.44-7.79)
*b*_4_	Exp(100)	3.00	2.85 (0.35-7.19)	2.64 (1.82-3.32)

Parameter values used in simulation, prior distribution, posterior medians and 95% credibility intervals. Estimate 1 corresponds to inference from single time series, Estimate 2 relates to estimates obtained from 20 independent time series. Data used for inference are plotted in Figure A for case **A **and Figure B for case **B**. Variance of the measurement error was assumed to be known *σ*_ϵ _= 9. Rates are per hour. Parameters are *n*_*H *_= 1, *H *= 61.98 in case **B**.

Inference for this model required sampling from the 9 dimensional posterior distribution (number of unknown parameters). If instead one used a diffusion approximation based method it would be necessary to sample from a posterior distribution of much higher dimension (see Additional file 1). In addition, incorporation of the measurement error is straightforward here, whereas for other methods it involves a substantial computational cost [20].

### Inference from fluorescent reporter gene data for the model of single gene expression with autoregulation

The following example considers the autoregulatory system with only a small number of reacting molecules. Using SSA we generated artificial data from the single gene expression model with autoregulation. The protein time courses were then sampled every 15 minutes at 101 discrete points per trajectory (see Figure 1B). As before we assume that the mRNA time courses are not observed and that the protein data are of the form given in (20), i.e. proportional to the actual amount of protein with additive Gaussian measurement error. As in the previous case study we estimate parameters from two simulated data sets, a single trajectory and an ensemble of 20 independent trajectories. The inference results summarized in Table 1B show that despite the low number of mRNA (0-15 molecules, see Figure 2 in Additional file 1) and protein (10-250 molecules, see Figure B) all parameters can be estimated well with appropriate precision.

### Inference for PCR based reporter data

In the case of reporter assays based on Polymerase Chain Reaction (e.g. RT-PCR, Q-PCR) measurements are obtained from the extraction of the molecular contents from the inside of cells. Since the sample is sacrificed, the sequence of measurements are not strictly associated with a stochastic process describing the same evolving unit. Assume that at each time point *t*_*i *_(*i *= 0,..*n*) we observe *l *measurements that are proportional to the number of RNA molecules either from a single cell or from a population of *s *cells. This gives a (*n *+ 1) × *l *matrix of data points

(22)

where  is the actual RNA level, *λ *is the proportionality constant,  is a Gaussian independent measurement error indexed by time *t*_*i*_. *j *= 1,..., *l *indexes the *l *measurements that are taken at time *t*_*i*_. Note that  and  are independent random variables as they refer to different cells. We assume that the dynamics of RNA is described by the simple model of single gene expression with LNA equations (16) and (17). Let ϒ_*t *_denote the distribution of measured RNA at time *t *(*u*_*t *_~ ϒ_*t*_). In order to accommodate for the different form of data we modify the estimation procedure as follows. For analytical convenience we assumed that the initial distribution is normal . This together with eq. (8) and normality of measurement error implies that . Simple explicit formulae for *μ*_*t *_and  are derived in Additional file 1. Since all observations  are independent we can write the posterior distribution as

(23)

where *ψ*(·|, ) is the normal density with parameters , . In order to infer the vector of the unknown parameters Θ = (*γ*_*R*_, *λ*, *b*_0_, *b*_1_, *b*_2_, *b*_3_, *b*_4_, , ) we sample from the posterior using a standard MH algorithm. To test the algorithm we have simulated a small (*l *= 10, *n *= 50, plotted in Figure 2) and a large (*l *= 100, *n *= 50) data set using SSA algorithm with parameter values given in Table 2. The data were sampled discretely every 30 minutes and a standard normal error was added. Initial conditions were sampled from the Poisson distribution with mean *b*_4_/*γ*_*R*_. The estimation results in Table 2 show that parameters can be inferred well in both cases even though the number of RNA molecules in the generated data is very small (about 5-35 molecules). Since subsequent measurements do not belong to the same stochastic trajectory, estimation for the model presented here is not straightforward with the diffusion approximation based methods.

**Table 2:** Inference results for PCR based reporter assay simulated data

**Parameter**	**Prior**	**Value**	**Estimate 1**	**Estimate 2**
*γ*_*R*_	Exp(1)	0.44	0.45 (0.35-0.60)	0.46 (0.42-0.50)
*λ*	Exp(100)	1.00	1.07 (0.90-1.22)	1.01 (0.95-1.05)
*b*_0_	Exp(100)	15.00	13.13 (10.20-15.87)	14.91 (13.86-15.77)
*b*_1_	Exp(1)	0.40	0.29 (0.14-0.51)	0.43 (0.32-0.54)
*b*_2_	Exp(1)	0.40	0.32 (0.12-0.93)	0.32 (0.21-0.43)
*b*_3_	Exp(10)	7.00	7.05 (6.39-7.63)	6.99 (6.76-7.15)
*b*_4_	Exp(100)	3.00	2.97 (2.00-4.18)	3.10 (2.76-3.43)
*μ*_0_	Exp(100)	6.76	6.90 (5.79-7.69)	6.55 (6.14-6.85)
	Exp(100)	6.76	3.52 (0.01-8.99)	7.59 (5.44-9.49)

Parameter values used to generate data, prior distributions used for estimation, posterior median estimates together with 95% credibility intervals. Estimate 1, Estimate 2 columns relate to small (l = 5, n = 50) and large (l = 100, n = 50) sample sizes. Variance of the measurement was assumed to be known  = 4. Estimated rates are per hour.

### Estimation of gfp protein degradation rate from cycloheximide experiment

In this section the method is applied to experimental data. After a period of transcriptional induction, translation of gfp was blocked by the addition of cycloheximide (CHX). Details of the experiment are presented in Additional file 1. Fluorescence was imaged every 6 minutes for 12.5 h (see Figure 2). Since inhibition may not be fully efficient we assume that translation may be occurring at a (possibly small) positive rate *k*_*P*_. The model with the LNA is

(24)

The observed fluorescence is assumed to be proportional to the signal with proportionality constant *λ*. For comparison we also consider the diffusion approximation for which an exact transition density can be derived analytically (see Additional file 1 for derivation)

(25)

Since incorporation of measurement error for the diffusion approximation based model is not straightforward, we assume that measurements were taken without any error to ensure fair comparison between the two approaches. Table 3 shows that estimates obtained with both methods are not very different.

**Table 3:** Inference results for CHX experimental data

**Param**.	**Prior**	**Estimate LNA**	**Estimate DA**
*γ*_*P*_	Exp(1)	0.45 (0.31-0.62)	0.53 (0.39-0.67)
*k*_*P*_	Exp(50)	0.32(0.10-1.75)	0.43 (0.16-1.07)
*λ*	Exp(50)	22.79(13.79-36.92)	23.85(16.31-36.54)
		889.03(831.44-945.34)	-

Priors, posterior mean and 95% credibility intervals obtained from CHX experimental data using the LNA approach and diffusion approximation approach. Estimation with the LNA involved one more parameter . Estimated rates are per hour.

## Conclusion

The aim of this paper is to suggest the LNA as a useful and novel approach to the inference of biochemical kinetics parameters. Its major advantage is that an explicit formula for the likelihood can be derived even for systems with unobserved variables and data with additional measurement error. In contrast to the more established diffusion approximation based methods [9,20] the computationally costly methods of data augmentation to approximate transition densities and to integrate out unobserved model variables are not necessary. Furthermore, this method can also accommodate measurement error in a straightforward way.

The suggested procedure here is implemented in a Bayesian framework using MCMC simulation to generate posterior distributions. The LNA has previously been studied in the context of approximating Poisson birth and death processes [22-24,35] and it was shown that for a large class of models the LNA provides an excellent approximation. Furthermore, in [35] it is shown that for the systems with linear reaction rates the first two moments of the transition densities resulting from the CME and the LNA are equal. Here we propose using the LNA directly for inference and provide evidence that the resulting method can give very good results even if the number of reacting molecules is very small. In our previous study [10] we have presented differences between fitting deterministic and stochastic models, where we used diffusion approximation based method. Our experience from that work and from study [20] is that implementation of diffusion approximation based methods is challenging especially for data that are sparsely sampled in time because the need for imputation of unobserved time points leads to a very high dimensionality of the posterior distribution. This usually results in highly autocorrelated traces affecting the speed of convergence of the Markov chain. Our method considerably reduces the dimension of the posterior distribution to the number of unknown parameters of a model only and is independent of the number of unobserved components (see Additional file 1). Nevertheless it can only be applied to the systems with sufficiently large volume, where fluctuations around a deterministic state are relatively close to the mean.

## Authors' contributions

MK proposed and implemented the algorithm. CVH performed the cycloheximide experiment. MK wrote the paper with assistance from BF and DAR, who supervised the study.

## Supplementary Material

Additional file 1 **Supplemental information**. Supplementary information contains derivation of the theoretical results, details about algorithm implementation and comparison with the inference method based on the diffusion approximation. Click here for file
