# Supplementary material for: Bayesian inference of biochemical kinetic parameters using the linear noise approximation
Source: BMC Bioinformatics. 2009 Oct 19;10:343. doi: 10.1186/1471-2105-10-343 (PMC2774326; doi:10.1186/1471-2105-10-343)
Supplement: Additional file 1 — Supplemental information. Supplementary information contains derivation of the theoretical results, details about algorithm implementation and comparison with the inference method based on the diffusion approximation. [file 1471-2105-10-343-S1.PDF]

Supplementary Information

Bayesian inference of biochemical kinetic parameters using the linear noise approximation

Michał Komorowski[1,2], Bärbel Finkenstädt[1], Claire V. Harper[4], David A. Rand[2,3]

1. Department of Statistics, University of Warwick, Coventry, UK
2. Systems Biology Centre, University of Warwick, Coventry, UK
3. Mathematics Institute, University of Warwick, Coventry, UK
4. Department of Biology, University of Liverpool, Liverpool, UK

This is supplementary information for the paper *Bayesian inference of biochemical kinetic parameters using a linear noise approximation* which is henceforth referred to as **I**.

1 Modelling of Chemical Kinetics

In this section we derive the macroscopic rate equation (MRE), the diffusion approximation (DA) and the linear noise approximation (LNA) for the chemical system described in the section **Methods** of the main paper **I**. Our derivations in section 1 follow [1] and [2] . The chemical master equation (CME) describes the time evolution of the probability h that at time t the system is in the state $\mathbf{X}$

$$\frac{dh(\mathbf{X},t)}{dt} = \Omega \sum_{j=1}^{R} \left(\prod_{i=1}^{N} E^{-S_{ij}} - 1 \right) \tilde{f}_j(\mathbf{x},\Omega,t) h(\mathbf{X},t). \tag{1}$$

Here, $E^{-S_{ij}}$ is a step operator defined by

$$E^{-S_{ij}} f(...,X_i,...) = f(...,X_i - S_{ij},...).$$

1.1 Macroscopic rate equation

As the system's volume Ω increases, relative fluctuations become negligible and in the limit of infinitely large Ω the system becomes deterministic. To derive the macroscopic rate equation we write the operator $\prod_{i=1}^{N} E^{-S_{ij}}$ in the form of a first order multivariate Taylor expansion

$$\prod_{i=1}^{N} E^{-S_{ij}} = 1 - \sum_{i=1}^{N} \frac{S_{ij}}{\Omega} \frac{\partial}{\partial x_i} + O(\Omega^{-2}).$$

After substitution into the CME (1), in the limit of infinitely large Ω we obtain

$$\frac{dh(\varphi,t)}{dt} = \sum_{j=1}^{R}\left(\sum_{i=1}^{N} S_{ij}\frac{\partial}{\partial\phi_i}\right) f_j(\varphi,t)h(\varphi,t). \tag{2}$$

This partial differential equation can be solved by the method of characteristics. The solution is called the *macroscopoic rate equation* and has the form

$$\frac{d\phi_i}{dt} = \sum_{j=1}^{R} S_{ij} f_j(\varphi,t) \qquad i = 1,2,...,N. \tag{3}$$

1.2 Diffusion approximation

Similarly, one may write the second order Taylor approximation of the step operator in the following way

$$\prod_{i=1}^{N} E^{-S_{ij}} = 1 - \sum_{i=1}^{N}\frac{S_{ij}}{\Omega}\frac{\partial}{\partial x_i} + \frac{1}{2}\frac{1}{\Omega^2}\sum_i\sum_k S_{ij}S_{ik}\frac{\partial^2}{\partial x_i \partial x_k} + O(\Omega^{-3}).$$

Again, if the volume is large enough the terms of order $O(\Omega^{-3})$ can be neglected and substitution of the expanded operator into (1) implies the Fokker-Planck equation of the form

$$\frac{dh(\mathbf{x},t)}{dt} = -\sum_{i=1}^{N}\sum_{k=1}^{R}\frac{\partial}{\partial x_i}[\mathbf{A}]_{ik}h(\mathbf{x},t) + \frac{1}{2}\sum_{i,k=1}^{N}\frac{\partial}{\partial x_i}\frac{\partial}{\partial x_k}\left[\mathbf{E}\mathbf{E}^T\right]_{ik} h(\mathbf{x},t), \tag{4}$$

where

$$\begin{aligned}
[\mathbf{A}]_{ik} &= S_{ik}\tilde{f}_k(\mathbf{x},\Omega,t),\ \mathbf{E} = \frac{1}{\sqrt{\Omega}}S\sqrt{diag(\tilde{\mathbf{f}}(\mathbf{x},\Omega,t))},\\
\left[\mathbf{E}\mathbf{E}^T\right]_{ik} &= \sum_{j=1}^{R}\frac{1}{\Omega}S_{ij}S_{kj}\tilde{f}_j(\mathbf{x},\Omega,t)\\
\tilde{\mathbf{f}}(\mathbf{x},\Omega,t) &= (\tilde{f}_1(\mathbf{x},\Omega,t),...,\tilde{f}_R(\mathbf{x},\Omega,t))^T.
\end{aligned}$$

The above Fokker-Planck equation describes the time evolution of the transition densities of the Itô diffusion equation [3]

$$d\mathbf{x} = \mathbf{A}(\mathbf{x},\mathbf{t})dt + \mathbf{E}(\mathbf{x},\mathbf{t})dW, \tag{5}$$

where dW denotes increments of the Wiener process.

1.3 Linear noise approximation

In order to obtain the linear noise approximation transition rates, $\tilde{f}_j(\mathbf{x}, t)$ and the step operator $E^{\cdot}$ are Taylor expanded around the deterministic state φ in powers of $1/\sqrt{\Omega}$. To obtain such an expansion process, X_i is decomposed into the deterministic φ and stochastic $\xi = (\xi_1, ..., \xi_N)^T$ components according to the relation

$$X_i \equiv \Omega\phi_i + \Omega^{1/2}\xi_i. \tag{6}$$

Transition rates are expanded as follows

$$\tilde{f}_j(\mathbf{x}, t) = f_j(\varphi, t) + \frac{1}{\sqrt{\Omega}} \sum_{i=1}^{N} \frac{\partial f_i(\varphi, t)}{\partial \phi_i} \xi_i + O(\Omega^{-1}). \tag{7}$$

Similarly, we have an expansion of the step operator

$$\prod_{i=1}^{N} E^{-S_{ij}} = 1 - \Omega^{-1/2} \sum_{i=1}^{N} S_{ij} \frac{\partial}{\partial \xi_i} + \frac{1}{2\Omega} \sum_{i=1}^{N} \sum_{k=1}^{N} S_{ij} S_{kj} \frac{\partial^2}{\partial \xi_i \partial \xi_k} + O(\Omega^{-\frac{3}{2}}). \tag{8}$$

Let us denote the probability distribution of ξ at time t by $\Pi(\xi, t)$. Using the fact that the distribution $h(\mathbf{X}, t)$ is related to $\Pi(\xi, t)$ through the relation

$$h(\mathbf{X}, t) = h(\Omega\varphi + \Omega^{1/2}\xi, t) = \Pi(\xi, t) \tag{9}$$

and putting (7), (8) and 9 into (1), we obtain the Fokker-Planck equation describing the evolution of Π [2]

$$\frac{d\Pi(\xi, t)}{dt} = -\sum_{i,k=1}^{N} [\mathbf{A}]_{ik} \frac{\partial}{\partial \xi_i} \xi_k \Pi + \frac{1}{2} \sum_{i,k=1}^{N} \left[\mathbf{E}\mathbf{E}^T\right]_{ik} \frac{\partial^2 \Pi}{\partial \xi_i \partial \xi_k}, \tag{10}$$

where

$$f_i = f_i(\varphi, t), \; [\mathbf{A}]_{ik} = \sum_{j=1}^{R} S_{ij} \frac{\partial f_j}{\partial \phi_k}, \; \mathbf{E} = S\sqrt{diag(\mathbf{f}(\varphi, t))}, \text{and } \left[\mathbf{E}\mathbf{E}^T\right]_{ik} = \sum_{j=1}^{R} S_{ij} S_{kj} f_j. \tag{11}$$

The related Itô diffusion equation has the form

$$d\xi(t) = \mathbf{A}(t)\xi dt + \mathbf{E}(t) dW. \tag{12}$$

It is a linear SDE with time inhomogeneous coefficients and its explicit solution has the form (4) in **I**.

2 Derivation of the likelihood function

In this section we derive the likelihood function (11) from **I**. We use the notation introduced in the section **Methods** of **I**.

Recall that in **I** we partitioned the process $\mathbf{x}_t$ into observed variables $\mathbf{y}_t$ and unobserved latent variables $\mathbf{z}_t$. The Markov property of the process $\mathbf{x}_t$ implies that the augmented likelihood function $P(\bar{\mathbf{y}}, \bar{\mathbf{z}}|\Theta)$ can be written as

$$P(\bar{\mathbf{y}}, \bar{\mathbf{z}}|\Theta) = \prod_{i=1}^{n} \mathbf{p}(\mathbf{x}_{t_i}|\mathbf{x}_{t_{i-1}}, \Theta)\mathbf{p}(\mathbf{x}_{t_0}|\Theta), \tag{13}$$

where $\mathbf{p}(\mathbf{x}_{t_i}|\mathbf{x}_{t_{i-1}}, \Theta) = \psi(\mathbf{x}_{t_i}|\varphi(t_i)+\Omega^{-\frac{1}{2}}\mu_{i-1}, \Omega^{-1}\Xi_{i-1})$ and $\mathbf{p}(\mathbf{x}_{t_0}|\Theta) = \psi(\mathbf{x}_{t_o}|\varphi(t_o), \Omega^{-1}\Xi_{-1})$. From now on to simplify notation we write μ_{i-1} instead of $\Omega^{-\frac{1}{2}}\mu_{i-1}$ and Ξ_{i-1} instead of $\Omega^{-1}\Xi_{i-1}$.

Eq. (2), (6) and (7) from **I** imply that $\mathbf{x}_{t_i}$ can be represented as

$$\mathbf{x}_{t_i} = \phi_{t_i} + \sum_{j=0}^{i} \Phi_{t_j}(t_i - t_j)\zeta_{t_j}, \tag{14}$$

where ζ_{t_j} are independently normally distributed random variables with mean 0 and covariance matrix Ξ_{j-1}. This implies that

$$P(\bar{\mathbf{y}}, \bar{\mathbf{z}}|\Theta) = \psi(\bar{\mathbf{x}}|(\varphi_{t_0}, \ldots, \varphi_{t_n}), \hat{\Sigma}), \tag{15}$$

where the covariance matrix $\hat{\Sigma} = \{\hat{\Sigma}^{(i,j)}\}_{i,j=0,\ldots,n}$, is the $(n+1)N \times (n+1)N$ block matrix that is composed of $N \times N$ submatrices $\hat{\Sigma}^{(i,j)} = Cov(\mathbf{x}_{t_i}, \mathbf{x}_{t_j})$. Covariances $Cov(\mathbf{x}_{t_i}, \mathbf{x}_{t_j})$ can be computed using the following relations ($j \geq i$)

$$\begin{aligned} Cov(\mathbf{x}_{t_0}, \mathbf{x}_{t_0}) &= \Xi_{-1}, & (16)\\ Cov(\mathbf{x}_{t_i}, \mathbf{x}_{t_i}) &= \Xi_{i-1} + \Phi_{t_{i-1}}(\Delta_{i-1})Cov(\mathbf{x}_{t_{i-1}}, \mathbf{x}_{t_{i-1}})\Phi_{t_{i-1}}(\Delta_{i-1})^T, & (17)\\ Cov(\mathbf{x}_{t_i}, \mathbf{x}_{t_{j+1}}) &= Cov(\mathbf{x}_{t_i}, \mathbf{x}_{t_j})\Phi_{t_j}(\Delta_j)^T. & (18) \end{aligned}$$

The initial covariance matrix Ξ_{-1} can be either treated as a model parameter or expressed in term of other parameters.

In order to find the likelihood function $P(\bar{\mathbf{y}}|\Theta)$ from the augmented likelihood (15) we use the fact that marginal distributions of the normal distribution are normal. Thus, we obtain

$$P(\bar{\mathbf{y}}|\Theta) = \psi(\bar{\mathbf{y}}|(\varphi_y(t_0), \ldots, \varphi_y(t_n)), \Sigma), \tag{19}$$

where the covariance matrix Σ is a block matrix $\Sigma = \{\Sigma^{(i,j)}\}_{i,j=0,\ldots,n}$ and $\Sigma^{(i,j)} = Cov(\mathbf{y}_{t_i}, \mathbf{y}_{t_j})$. Therefore $\Sigma^{(i,j)}$ is the lower right square submatrix of $\hat{\Sigma}^{(i,j)}$ which corresponds to the observed part of the process.

3 Examples

3.1 The simple model of single gene expression

The simple model of single gene expression can be summarised by the following stoichiometric equations [4]

$$\begin{aligned}
\mathrm{R}_1 : DNA &\xrightarrow{k_R(t)} DNA + R \\
\mathrm{R}_2 : R &\xrightarrow{\gamma_R R/\Omega} \varnothing \\
\mathrm{R}_3 : R &\xrightarrow{k_P R/\Omega} R + P \\
\mathrm{R}_4 : P &\xrightarrow{\gamma_P P/\Omega} \varnothing
\end{aligned}$$

Vectors of molecular copy numbers ($\mathbf{X}$), concentrations ($\mathbf{x}$), and macroscopic counterparts are

$$\mathbf{X} = (R, P), \quad \mathbf{x} = (r, p), \quad \varphi = (\phi_R, \phi_P).$$

The mesoscopic and macroscopic transition rate vectors and stoichiometric matrix have the form

$$\tilde{\mathbf{f}}(\mathbf{x}, t) = \begin{pmatrix} k_R(t) \\ \gamma_R r \\ k_P r \\ \gamma_P p \end{pmatrix}, \quad \mathbf{f}(\varphi, t) = \begin{pmatrix} k_R(t) \\ \gamma_R \phi_R \\ k_P \phi_R \\ \gamma_P \phi_P \end{pmatrix}, \quad \mathbf{S} = \begin{pmatrix} 1 & -1 & 0 & 0 \\ 0 & 0 & 1 & -1 \end{pmatrix}. \tag{20}$$

3.1.1 Chemical master equation

To obtain the CME for the system we substitute (20) into (1) to obtain [5]

$$\begin{aligned}
&\frac{dh(R, P, t)}{dt} = \\
&\Omega k_R(t)(h(R-1, P, t) - h(R, P, t)) + k_P R(h(R, P-1, t) - h(R, P, t)) \\
&+\gamma_R(h(R+1, P, t)(R+1) - h(R, P, t)R) + \gamma_P(h(R, P+1, t)(P+1) - h(R, P, t)P).
\end{aligned} \tag{21}$$

3.1.2 Macroscopic rate equations

Similarly (3) and (20) results in

$$\begin{aligned}
\dot{\phi}_R &= k_R(t) - \gamma_R \phi_R, \\
\dot{\phi}_P &= k_P \phi_R - \gamma_P \phi_P.
\end{aligned} \tag{22}$$

3.1.3 Diffusion approximation

In order to apply diffusion approximation to CME (21) we need the drift $\mathbf{A}(\mathbf{x}, t)$ and diffusion matrices $\mathbf{E}\mathbf{E}^T$. By substitution of (20) into (4) we get

$$\mathbf{A}(t) = \begin{pmatrix} k_R(t) & -\gamma_R r \\ k_P r & -\gamma_P p \end{pmatrix}, \qquad (\mathbf{E}\mathbf{E}^T)(t) = \frac{1}{\Omega}\begin{pmatrix} k_R(t) + \gamma_R r & 0 \\ 0 & k_P\phi_R + \gamma_P p \end{pmatrix}, \tag{23}$$

where $E = S\sqrt{diag(\mathbf{f}(\varphi))}$. The above matrices imply the Fokker-Planck equation:

$$\begin{aligned} \frac{dh(r,p,t)}{dt} &= -\frac{\partial}{\partial r}(k_R(t) - \gamma_R r)h(r,p,t) \\ &- \frac{\partial}{\partial p}(k_P r - \gamma_P p)h(r,p,t) \\ &+ \frac{1}{2\Omega}\frac{\partial^2}{\partial r}(k_R(t) + \gamma_R r)h(r,p,t) \\ &+ \frac{1}{2\Omega}\frac{\partial^2}{\partial p}(k_P r + \gamma_P p)h(r,p,t). \end{aligned} \tag{24}$$

This corresponds to the Itô diffusion

$$\begin{aligned} dr &= (k_R(t) - \gamma_R r)dt + \sqrt{1/\Omega}\sqrt{k_R(t) + \gamma_R r}dW_r, \\ dp &= (k_P r - \gamma_P p)dt + \sqrt{1/\Omega}\sqrt{k_P p + \gamma_P P}dW_p. \end{aligned} \tag{25}$$

3.1.4 Linear noise approximation

In the LNA the deterministic and stochastic part are separated according to (6) so that $r(t) = \phi_R + \Omega^{-1/2}\xi_R$, $p(t) = \phi_P + \Omega^{-1/2}\xi_P$. In order to derive drift matrix $\mathbf{A}$ and diffusion matrix $\mathbf{E}\mathbf{E}^T$ we first calculate

$$\frac{\partial \mathbf{f}}{\partial \phi_R} = (0, \gamma_R, k_P, 0)^T,$$

$$\frac{\partial \mathbf{f}}{\partial \phi_P} = (0, 0, 0, \gamma_P)^T.$$

and substitute the above formulae together with (20) into (11). We obtain

$$\mathbf{A} = \begin{pmatrix} -\gamma_R & 0 \\ k_P & -\gamma_P \end{pmatrix}, \qquad \mathbf{E}(t) = \begin{pmatrix} \sqrt{k_R(t)} & -\sqrt{\gamma_R\phi_R(t)} & 0 & 0 \\ 0 & 0 & \sqrt{k_P\phi_R(t)} & -\sqrt{\gamma_P\phi_P(t)} \end{pmatrix}, \tag{26}$$

and

$$(\mathbf{EE}^T)(t) = \begin{pmatrix} k_R(t) + \gamma_R\phi_R & 0 \\ 0 & k_P r + \gamma_P\phi_P \end{pmatrix}. \tag{27}$$

Hence, the Fokker-Planck equation has the form

$$\begin{aligned} \frac{dh(\xi_R, \xi_P, t)}{dt} &= -\frac{\partial}{\partial \xi_R}(-\gamma_R\xi_R)h(\xi_R, \xi_P, t) \\ &- \frac{\partial}{\partial \xi_P}(k_P\xi_R - \gamma_P\xi_P)h(\xi_R, \xi_P, t) \\ &+ \frac{1}{2}\frac{\partial^2}{\partial \xi_R}(k_R(t) + \gamma_R\phi_R(t))h(\xi_R, \xi_P, t) \\ &+ \frac{1}{2}\frac{\partial^2}{\partial \xi_P}(k_P\xi_R + \gamma_P\phi_P(t))h(\xi_R, \xi_P, t) \end{aligned}$$

and implies the Itô diffusion

$$\begin{aligned} d\xi_R &= (-\gamma_R\xi_R)dt + \sqrt{k_R(t) + \gamma_R\phi_R}dW_{\xi_R}, \\ d\xi_P &= (k_P\xi_R - \gamma_P\xi_P)dt + \sqrt{k_P\phi_P + \gamma_P\phi_P}dW_{\xi_P}. \end{aligned} \tag{28}$$

Given matrices $\mathbf{A}$ and $\mathbf{E}$ we can derive the above equations directly from (12)

$$\begin{aligned} d\xi_R &= (-\gamma_R\xi_R)dt + \sqrt{k_R(t)}dW_1 + \sqrt{\gamma_R\phi_R}dW_2, \\ d\xi_P &= (k_P\xi_R - \gamma_P\xi_P)dt + \sqrt{k_P\phi_P}dW_3 + \sqrt{\gamma_P\phi_P}dW_4. \end{aligned}$$

This equations are equivalent to (28) as we can write

$$\begin{aligned} \sqrt{\phi_R(t)}dW_1 + \sqrt{\gamma_R\phi_R}dW_2 &= \sqrt{k_R(t) + \gamma_R\phi_R}dW_{\xi_R}, \\ \sqrt{k_P\phi_P}dW_3 + \sqrt{\gamma_P\phi_P}dW_4 &= \sqrt{k_P\phi_P + \gamma_P\phi_P}dW_{\xi_P}. \end{aligned}$$

We assume that before time t_0 the transcription rate was constant and equal $k_R(t_0)$ ($k_R(t) = k_R(t_0)$ for $t \leq t_0$) and that the system is in the stationary state at time t_0. Therefore the covariance matrix Ξ_{-1} is the covariance matrix of the stationary distribution of the process (28) and by the fluctuation-dissipation theorem [1] can be found as the solution of the following equation

$$\mathbf{A}\Xi_{-1} + \Xi_{-1}\mathbf{A}^T + \mathbf{EE}^T(t_0) = 0. \tag{29}$$

3.2 Single gene expression with autoregulation

For the model of single gene expression with autoregulation the stoichiometric equation remain unchanged. The mesoscopic and macroscopic transition rates vector are as follows

$$\tilde{\mathbf{f}}(\mathbf{x},t) = \begin{pmatrix} k_R(t,\phi_P) \\ \gamma_R r \\ k_P r \\ \gamma_P p \end{pmatrix}, \qquad \mathbf{f}(\varphi,t) = \begin{pmatrix} k_R(t,\phi_P) \\ \gamma_R \phi_R \\ k_P \phi_R \\ \gamma_P \phi_P \end{pmatrix}. \tag{30}$$

where $k_R(t,p) = k_R(t)/(1+(p/H)^{n_H})$.

To derive the LNA equations for this model we use formulae (30) and eq. (11) and write the drift and diffusion matrices as

$$\mathbf{A} = \begin{pmatrix} -\gamma_R & k'_R(t) \\ k_P & -\gamma_P \end{pmatrix}, \qquad (\mathbf{E}\mathbf{E}^T)(t) = \begin{pmatrix} k_R(t,\phi_P)+\gamma_R\phi_R & 0 \\ 0 & k_P r + \gamma_P \phi_P \end{pmatrix}, \tag{31}$$

where $k'_R(t) = \partial k_R/\partial \phi_P(t,\phi_P)$. Therefore, the equations given by the LNA are as follows

$$\begin{aligned} \dot{\phi}_R &= k_R(t,\phi_P) - \gamma_R\phi_R, & (32)\\ \dot{\phi}_P &= k_P\phi_R - \gamma_P\phi_P, \\ d\xi_R &= (k'_R(t)\xi_P - \gamma_R\xi_R)dt + \sqrt{k_R(t)+\gamma_R\phi_R(t)}dW_R, \\ d\xi_P &= (k_P\xi_R - \gamma_P\xi_P)dt + \sqrt{k_P\phi_R(t)+\gamma_P\phi_P(t)}dW_P. & (33) \end{aligned}$$

Using the same argument as in the previous example we find the initial covariance matrix Ξ_{-1} as the solution of the following equation

$$\mathbf{A}(t_0)\Xi_{-1} + \Xi_{-1}\mathbf{A}(t_0)^T + \mathbf{E}(t_0)\mathbf{E}(t_0)^T = 0. \tag{34}$$

3.3 Derivation of likelihood for PCR based reporter data

In this section we derive formula (23) in **I**. The data for the PCR based reporter case has the form

$$\bar{\mathbf{u}} = \begin{pmatrix} u_{t_0,1} & u_{t_0,2} & ,\ldots, & u_{t_0,l-1} & u_{t_0,l} \\ u_{t_1,1} & u_{t_1,2} & ,\ldots, & u_{t_1,l-1} & u_{t_1,l} \\ \vdots & & & & \vdots \\ u_{t_{n-1},1} & u_{t_{n-1},2} & ,\ldots, & u_{t_{n-1},l-1} & u_{t_{n-1},l} \\ u_{t_n,1} & u_{t_n,2} & ,\ldots, & u_{t_n,l-1} & u_{t_n,l} \end{pmatrix}, \tag{35}$$

where $u_{t_i,j} = \lambda r_{t_i,j} + \epsilon_{t_i,j}$, $r_{t_i,j}$ is the actual RNA concentration, λ is the proportionality constant, $\epsilon_{t_i,j}$ is the normally and independently distributed measurement error with variance

σ_ϵ^2. The first of the lower indices t_i denotes the time of observation and the second index j refers to the measurement. The random variables $u_{t_i,j}$ and $u_{t_{i+1},j'}$ are independent since they belong to the different cells.

We set $\Omega = 1$ and assume that the RNA levels in all cells are described by independent processes $r(t) = \phi_R(t) + \xi_R(t)$, where

$$\begin{aligned}\dot{\phi}_R &= k_R(t) - \gamma_R\phi_R,\\ d\xi_R &= (-\gamma_R\xi_R)dt + \sqrt{k_R(t) + \gamma_R\phi_R}dW_{\xi_R}.\end{aligned}$$

We assume that $r(t_0)$ is normally distributed with mean $\tilde{\mu}_{t_0} = \phi_R(t_0)$ and variance $\tilde{\sigma}_{t_0}^2$. Using equations (7) and (8) of **I** we obtain that

$$\mathbf{p}(r(t)|\Theta) = \psi(r(t)|\phi_R(t), \tilde{\sigma}_t^2), \tag{36}$$

where

$$\tilde{\sigma}_t^2 = \int_{t_0}^{t} (exp(-2\gamma_R(t-s))((k_R(s) + \gamma_R\phi_R(s))))ds + \tilde{\sigma}_{t_0}^2 exp(-2(\gamma_R(t-t_0))). \tag{37}$$

Taking into account that $u_{t_i,j} = \lambda r_{t_i,j} + \epsilon_{t_i,j}$ we obtain that

$$\mathbf{p}(u_{t_i,j}|\Theta) = \psi\left(u_{t_i,j}|\mu_{t_i}, \sigma_{t_i}^2\right), \tag{38}$$

where

$$\mu_{t_i} = \lambda\phi_R(t_i), \qquad \sigma_{t_i}^2 = \lambda^2\tilde{\sigma}_{t_i}^2 + \sigma_\epsilon^2. \tag{39}$$

Since all observations are independent the likelihood function $P(\bar{\mathbf{u}}|\theta)$ has the form

$$P(\bar{\mathbf{u}}|\theta) = \prod_{i=0}^{n}\prod_{j=1}^{l}\psi(u_{t_i,j}|\mu_{t_i}, \sigma_{t_i}^2).$$

3.4 Cycloheximide experiment

Cycloheximide is an inhibitor of protein biosynthesis in eukaryotic organisms. It is widely used to determine degradation rates of proteins. In the experiment GH3 rat pituitary cells stably transfected with 5kb human prolactin promoter destabilised EGFP reporter construct (hPRL-d2EGFP) were seeded onto 35 mm glass coverslip-based dishes (IWAKI, Japan) and cultured in 10% FCS for 24h prior to imaging. Cells were transferred to the stage of a Zeiss Axiovert 200 equipped with an XL incubator (maintained at 37C, 5% CO2, in humid conditions) and images were obtained using a Fluar x20, 0.75 numerical aperture (Zeiss),

air objective. Excitation of d2EGFP was performed using an Argon ion laser at 488nm. Emitted light was captured through a 505-550 nm bandpass filter from a 545 nm dichroic mirror. Images were captured every 6 min. 5 μM forskolin and 0.5 μM BayK 8644 was added directly to the dish for 6h followed by the addition of 10μg/ml cyclohexamide to inhibit translation. Data was captured and analysed using LSM510 software with consecutive autofocus. Analysis was performed using Kinetic Imaging software AQM6. Regions of interest were drawn around each single cell and mean intensity data was collected over 14h.

We assume that action of cyclohexomide does not fully block translation but reduces the translation rate significantly. If the amount of mRNA is assumed constant then translation events can be treated as occurring at a small constant rate k_P. Then the model of single gene expression reduces to equations describing the variation in the amount of protein. From (22),(28) these are given by

$$\begin{aligned} \dot{\phi}_P &= k_P - \gamma_P \phi_P, \\ d\xi_P &= -\gamma_P \phi_P + \sqrt{k_P + \gamma_P \phi_P} dW_P. \end{aligned} \tag{40}$$

The DA can be used to obtain an analogous model. Again, neglecting fluctuation of mRNA concentration, assuming constant translation and setting $\Omega = 1$ from (25) we have

$$dp = (k_P - \gamma_P p)dt + \sqrt{k_P + \gamma_P p} dW_P. \tag{41}$$

By multiplication of the above equation with the scaling factor λ we obtain an equation for data $q = \lambda p$ proportional to the number of molecules

$$dq = (\lambda k_P - \gamma_P q)dt + \sqrt{\lambda}\sqrt{\lambda k_P + \gamma_P q} dW_P. \tag{42}$$

This equation is equivalent to Cox, Ingersoll and Ross model and has known transition densities [6] given by

$$\mathbf{p}(q_{t_{i+1}}|q_{t_i}) = \gamma_P c \exp(-u - v)(\frac{v}{u})^{\frac{w}{2}} I_w(2\sqrt{uv}), \tag{43}$$

where $c = 2(\lambda\gamma_P(1 - exp(-\gamma_P \Delta_{t_i})))^{-1}$, $u = c(\lambda k_P + \gamma_P p^M_{t_i}) \exp(-\gamma_P \Delta_{t_i})$, $v = c(\lambda k_P + \gamma_P p^M_{t_i})$, $w = \frac{4k_P}{\gamma_P} - 1$, $\Delta_{t_i} = t_{i+1} - t_i$ and $I_w(\cdot)$ is the modified Bessel function of the first kind of order w.

4 Validity of the LNA

In this section we provide some guidelines for decisions about whether our method can be used to obtain reliable estimates of kinetic rates or whether a more accurate method (e.g. DA) should be used.

The linear noise approximation has been obtained by Taylor expansion of the CME and reaction rates around deterministic system trajectories in terms of $1/\sqrt{\Omega}$. The rationale behind this expansion is that for constant average concentrations relative fluctuations will decrease with the inverse of the square root of the volume. Therefore the LNA is accurate when fluctuations are sufficiently small in relation to the mean (indication of large Ω). Hence, a natural measure of adequacy of the LNA is the ratio of the standard deviation to the mean, i.e. the coefficient of variation (CV). To clarify this principle consider again the simple model of single gene expression given by the CME (21). For simplicity assume that the transcription rate $k_R(t) = k_R$ is time-independent. It can be shown [4] that CVs for mRNA and protein concentrations have the form

$$CV(r) = \frac{1}{\sqrt{\Omega}} \frac{1}{\sqrt{k_R/\gamma_R}}, \qquad CV(p) = \frac{1}{\sqrt{\Omega}} \frac{\sqrt{1 + k_P/(\gamma_R + \gamma_P)}}{\sqrt{k_R k_P/\gamma_R \gamma_P}}. \tag{44}$$

The CV decreases with the $\sqrt{\Omega}$. Since Ω is not identifiable with k_R it can not be estimated from the data. Nevertheless, the CV can be easily calculated during the estimation procedure, since variances and means at all times t_i are computed to evaluate the likelihood function (19). Figure 1 presents the CV for mRNA and protein for the simple model of single gene expression and the model of single gene expression with autoregulation. The CV is always smaller than approximately 0.5 and decreases during times when the number of molecules is high. Our simulations show (data not presented) that for higher values of CV estimates may start to exhibit bias. Therefore for large values of the CV the LNA is likely to be a less reliable inference method.

There are two additional arguments that justify the usage of the LNA in a more precise way. If X is a Poisson birth and death process governed by the CME (1), φ is a solution of the MRE (3) and ξ is described by (12) then

1. the process $\Omega^{\frac{1}{2}}(\mathbf{X} - \Omega\varphi)$ weakly converges to the diffusion (12) as $\Omega \to \infty$ [7]; and
2. for the systems with linear reaction rates the mean and variance of transition densities of the process X and of the process $\Omega\varphi + \Omega^{1/2}\xi$ are equal [8].

5 Notes on the practical implementation of the algorithm

5.1 Computation of the likelihood

Computation of the likelihood function 12 in **I** can be summarised by the following steps

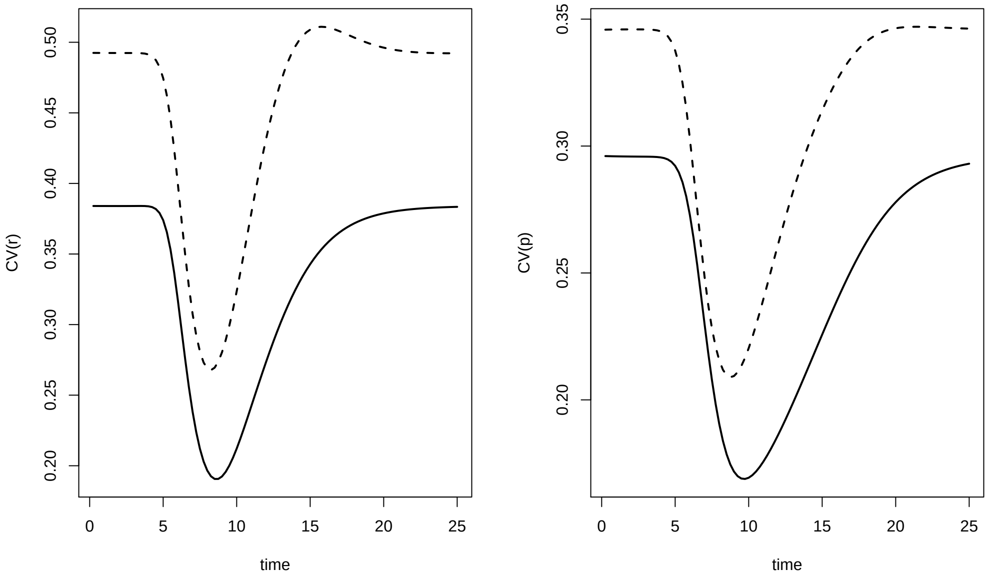

Figure 1: Coefficient of variation of RNA (left panel) and protein (right panel) for the models of simple gene expression (solid line) and gene expression with autoregulation (dashed line). The coefficient is calculated numerically for parameters presented in Tables 1A and 2B in **I**.

1 `Numerically find` $\varphi(t)$ `for` $t \in [t_0, t_n]$ `;`

2 `For` $i = 0, ..., n-1$ `find numerically fundamental matrices` $\Phi_{t_i}(s)$ `for` $s \in [t_i, t_{i+1}]$`;`

3 `Use results of steps 1 and 2 to compute covariance matrices` Ξ_{i-1} `for` $i = 0, ...n$`;`

4 `Use matrices computed in steps 2 and 3 to construct covariance matrix` $\hat{\Sigma}$ `according to the procedure from section 2;`

5 `Extract covariance matrix` Σ `from` $\hat{\Sigma}$ `(according to section 2);`

6 `For given data` $\bar{\mathbf{u}}$ `evaluate multivariate normal density with mean vector` $\lambda(\varphi(t_0), ..., \varphi(t_{n-1}))$ `and covariance matrix` $\lambda^2\Sigma + \Sigma_\epsilon$`, where` λ `and` Σ_ϵ `are defined the in section` **Methods** `of the` **I**`;`

5.2 Updating Θ

Since biochemical rates are positive it is convenient to parametrise the model in terms of logarithms of the original parameters. We denote the new parameterization by $\bar{\Theta} = (\bar{\theta}_1, ..., \bar{\theta}_k) = (log(\theta_1), ..., log(\theta_k))$. The posterior distributions $\hat{P}(\bar{\Theta}|\bar{\mathbf{u}})$ can be obtained from $P(\bar{\Theta}|\bar{\mathbf{u}})$ according to the reparameterization rule [9] given here by the equation

$$\hat{P}(\bar{\Theta}|\bar{\mathbf{u}}) = P(\Theta|\bar{\mathbf{u}}) \prod_{j=1}^{k} \theta_j \tag{45}$$

where $\prod_{j=1}^{k} \theta_j$ is a determinant of the inverse Jacobian matrix of the parameterization $\bar{\Theta}$. Each parameter $\bar{\theta}_j$ is updated individually using a random-walk Metropolis algorithm. Let $\bar{\theta}_j^{(i)}$ be the value of $\bar{\theta}_j$ at iteration i of the MCMC algorithm. A new value $\bar{\theta}_j^{(new)}$ is proposed from the symmetric proposal distribution

$$\bar{\theta}_j^{(new)} \sim N(\bar{\theta}_j^{(i)}, \sigma^2_{\theta_j})).$$

The new value $\bar{\theta}_j^{(new)}$ is then accepted with probability given by the following

$$\min\left\{1, \frac{\hat{P}(\bar{\Theta}_j^{(new)}, \bar{\Theta}_{-j,i}|\bar{\mathbf{u}})}{\hat{P}(\bar{\Theta}_j^{i}, \bar{\Theta}_{-j,i}|\bar{\mathbf{u}}))}\right\},$$

where $\bar{\Theta}_{-j,i}$ indicates all other parameters excluding $\bar{\theta}_j$ at iteration i and $\hat{P}(\bar{\Theta}, \bar{\mathbf{z}}|\bar{\mathbf{y}})$ is the posterior distribution of parameters $\bar{\Theta}$. If $\bar{\theta}_j^{(new)}$ is not accepted then $\bar{\theta}_j^{(i+1)} = \bar{\theta}_j^{(i)}$. The variance parameter of the proposal distribution, $\sigma^2_{\theta_j}$, is carefully chosen to ensure that the proposed moves are not too small (in this case there is very high acceptance of the proposed values and the chains take a long time to explore the parameter space) or too large (in this case the chains can get 'stuck' as the proposed parameter values are not often accepted which also leads to a slow exploration of the parameter space).

5.3 Numerical approximation of fundamental matrices

Consider the linear ODE

$$\frac{d\Phi_s}{dt} = \mathbf{A}(s+t)\Phi_s, \tag{46}$$

where $\mathbf{A}(s+t)$ and Φ_s is an $N \times N$ matrix. Let $\Phi_s(t)$ be the solution of this with initial condition the identity matrix i.e. $\Phi_s(0) = I$. In order to compute the transition density

covariances Ξ_{i-1} (eq. (7) in **I**), it is necessary to find these matrices. This can be done either by solving the equation directly (which gives $\Phi_s(t)$ as t varies) or by solving the adjoint equation (which gives $\Phi_s(t)$ as s varies). More detailed explanation can be found in [10].

6 Inference using diffusion approximation compared with the linear noise approximation based method

In this section we briefly describe inference methods based on the diffusion approximation. We also use the example of the simple model of gene expression to demonstrate advantages of using our method instead.

Similarly as in the section 1 of **I**. suppose we observe a discretely sampled multivariate time series $\bar{\mathbf{x}} = (x_{t_0}, ..., x_{t_n})$ that is assumed to be a realisation of the process (5). For simplicity we assume that all components of $\mathbf{x}$ are observed and are measured without error. The aim is to estimate the unknown parameters θ given the data $\bar{\mathbf{x}}$ through the posterior distribution $P(\theta|\bar{\mathbf{x}}) \propto P(\bar{\mathbf{x}}|\theta)\pi(\theta)$, where $\pi(\theta)$ denotes the prior distribution. In order to perform inference the likelihood $P(\bar{\mathbf{x}}|\theta)$ must be derived. Through the Markov property of the process (5) we have that

$$P(\bar{\mathbf{x}}|\theta) = \prod_{i=1}^{n} \mathbf{p}(\mathbf{x}_{t_i}|\mathbf{x}_{t_{i-1}}, \Theta). \tag{47}$$

Exact transition densities of the diffusion (5) are unknown and approximation have to be considered. If the time increment between observations $\Delta_{t_{i-1}} = t_i - t_{i-1}$ is small then a good approximation is given by the normal density [11]

$$\mathbf{p}(\mathbf{x}_{t_i}|\mathbf{x}_{t_{i-1}}) = \psi(\mathbf{x}_{t_i}|\mu_{t_{i-1}}, \Xi_{t_{i-1}}), \tag{48}$$

The mean $\mu_{t_{i-1}}$ and covariance matrix $\Xi_{t_{i-1}}$ are given by

$$\begin{aligned} \mu_{t_{i-1}} &= \mathbf{x}_{t_{i-1}} + \mathbf{A}(\mathbf{x}_{\mathbf{t_{i-1}}}, \mathbf{t_{i-1}})\Delta_{t_{i-1}}, & (49)\\ \Xi_{t_{i-1}} &= \Delta_{t_{i-1}}\ \mathbf{E}(\mathbf{x}_{\mathbf{t_{i-1}}}, \mathbf{t})\mathbf{E}(\mathbf{x}_{\mathbf{t_{i-1}}}, \mathbf{t})^T, & (50) \end{aligned}$$

where $\Delta_{t_{i-1}} = t_i - t_{i-1}$. Justification for this approximations follow from the Euler-Maruyama approximation of equation (5) and is discussed in details in [11].

In practical applications the Δ_{t_i} are usually not small. There exist various approaches in the literature to deal with such a situation (e.g. [12],[6],[13]). One simple idea leading to

MCMC based inference is to augment the data by introducing a finer set of times $\tau_{i,j}$ so that each interval $[t_i, t_{i+1}]$ is partitioned into $M+1$ subintervals $[t_i = \tau_{i,0}, \tau_{i,1}, ..., , \tau_{i,M+1} = t_{i+1}]$. Data is imputed at the new times $\tau_{i,j}$ which we will denote by $x^*_{\tau_{i,j}}$, $j = 1, ..., M$. Let denote $\bar{\mathbf{x}}^*$ the set of all imputed points.

The new times are chosen so that the Euler approximation can be safely assumed to be accurate on each subinterval $[\tau_{i,j}, \tau_{i,j+1}]$. We can then use equation (47) to obtain an augmented approximate likelihood $P(\bar{\mathbf{x}}, \bar{\mathbf{x}}^*|\theta)$ and write densities $\mathbf{p}(\mathbf{x}_{t_i}|\mathbf{x}_{t_{i-1}})$ in terms of imputed variables $\mathbf{x}^*_{\tau_{i-1,1}}, ..., \mathbf{x}^*_{\tau_{i-1,M}}$

$$\mathbf{p}(\mathbf{x}_{t_i}|\mathbf{x}_{t_{i-1}}) = \prod_{j=0}^{M+1} \mathbf{p}(\mathbf{x}^*_{\tau_{(i-1)j}}|\mathbf{x}^*_{t_{(i-1)j}}) \tag{51}$$

and $\mathbf{p}(\mathbf{x}_{\tau_{(i-1)j}}|\mathbf{x}_{\tau_{(i-1)j}})$ are calculated according to the formula (48). Monte Carlo methods provide a feasible way to integrate out auxiliary variables.

By Bayes' theorem, $P(\theta, \bar{\mathbf{x}}^*|\bar{\mathbf{x}}) \propto P(\bar{\mathbf{x}}^*, \bar{\mathbf{x}}|\theta)\pi(\theta)$. Thus, to provide an estimate of θ from sparsely sampled data, MCMC can be used to sample from the joint posterior $P(\theta, \bar{\mathbf{x}}^*|\bar{\mathbf{x}})$ of the parameters θ and the auxiliary variables $\bar{\mathbf{x}}^*$ given the data $\bar{\mathbf{x}}$. The main problem of this approach is that it increases the dimension of posterior distribution by nMN (number of imputed points). The high dimension of a posterior distribution leads to highly correlated Markov Chains. Therefore long chains must be generated to provide a reliable sample from the posterior distribution. It may become practically unfeasible or extremely difficult if the data frequency is low (large M needed) or if the process $\mathbf{x}$ is high-dimensional.

If some components of the process $\mathbf{x}$ are unobserved then the same data augmentation procedure may be used to integrate out unobserved variables.

6.1 Inference for single gene expression model using the diffusion approximation

To illustrate problems related to inference using the diffusion approximation method we use the simple model of single gene expression (More detailed explanation can be found in [14]). Suppose we have a sequence of measurements

$$\bar{\mathbf{x}} = (p_{t_0}, p_{t_1}, ..., p_{t_n}),$$

that can be treated as a realisation of the p component of the process (25). Assume that $\Omega = 1$. To perform inference between each pair of subsequent observations $(p_{t_i}, p_{t_{i+1}})$ M additional points are introduced. In addition the r process in unobserved. Therefore the

augmented data matrix (matrix composed of both $\bar{\mathbf{x}}^*$ and $\bar{\mathbf{x}}$) has the form

$$\begin{pmatrix} r^*_{t_0} & r^*_{\tau_{0,1}} & \cdots & r^*_{\tau_{0,M}} & r^*_{t_1} & \cdots & r^*_{t_{n-1}} & r^*_{\tau_{n-1,1}} & \cdots & r^*_{\tau_{n-1,M}} & r^*_{t_n} \\ p_{t_0} & p^*_{\tau_{0,1}} & \cdots & p^*_{\tau_{0,M}} & p_{t_1} & \cdots & p_{t_{n-1}} & p^*_{\tau_{n-1,1}} & \cdots & p^*_{\tau_{n-1,M}} & p_{t_n} \end{pmatrix}.$$

There are $2n(M+1)+2$ elements of the augmented data matrix (of which $2nM+n+1$ are unknown and $n+1$ are known). Therefore, the posterior distribution $P(\bar{\mathbf{x}}^*, \theta|\bar{\mathbf{x}}^*)$ has dimension $2n(M)+n+1+\dim\Theta$.

For comparison, if we use the approach based on the LNA the number of unknowns is equal to the dimension of Θ.

Practical adjustment of the parameter M depends mostly on the time distance between observations. For instance, if we assume that in an experiment fluorescence is measured every 17 minutes, 101 times in total (n=100) and if we set $M = 15$, postulating that RNA and protein changes in one minute intervals are normal, then we obtain that the dimension of the posterior equals 3101 plus number of elements of the vector Θ. If we use the LNA based approach, proposed in this paper, the analogous posterior has the dimension equal to the dimension of Θ.

References

1. N.G. Van Kampen. *Stochastic Processes in Physics and Chemistry.* North Holland, 2006.

2. J. Elf and M. Ehrenberg. Fast Evaluation of Fluctuations in Biochemical Networks With the Linear Noise Approximation. *Genome Res.*, 13(11):2475–2484, 2003.

3. C. Gardiner. *Handbook of stochastic methods.* Springer, 1985.

4. M. Thattai and A. van Oudenaarden. Intrinsic noise in gene regulatory networks. *Proceedings of the National Academy of Sciences*, page 151588598, 2001.

5. M. Komorowski, J. Miekisz, and A. Kierzek. Translational Repression Contributes Greater Noise to Gene Expression than Transcriptional Repression. *Biophysical Journal*, 96(2), 2009.

6. G.B. Durham and Gallant A.R. Numerical techniques for maximum likelihood estimation of continuous-time diffusion processes. *Journal of Business and Economic Statistics*, 20:297–316, 2002.

7. T.G. Kurtz. The Relationship between Stochastic and Deterministic Models for Chemical Reactions. *The Journal of Chemical Physics*, 57(7):2976–2978, 1972.

8. R. Tomioka, H. Kimura, T. J. Kobayashi, and K. Aihara. Multivariate analysis of noise in genetic regulatory networks. *Journal of Theoretical Biology*, 229(4):501–521, 2004.

9. D. Gamerman and H. F. Lopes. *Markov Chain Monte Carlo Stochastic Simulation for Bayesian Inference, 2nd ed.* Chapman & Hall/CRC, 2006.

10. D. Zwillinger. *Handbook of Differential Equations.* San Diego, 1989.

11. P. E. Kloeden and Platen E. *Numerical Solution of Stochastic differential equations.* Springer, 1999.

12. O. Elerian, S. Chib, and N. Shephard. Likelihood inference for discretely observed nonlinear diffusions. *Econometrica*, 69(4):959–993, 2001.

13. A. Beskos, O. Papaspiliopoulos, G. O. Roberts, and P. Fearnhead. Exact and computationally efficient likelihood-based estimation for discretely observed diffusion processes (with discussion). *Journal of the Royal Statistical Society: Series B (Statistical Methodology)*, 68(3):333–382, 2006.

14. B. Finkenstadt, E.A. Heron, M. Komorowski, K. Edwards, S. Tang, C.V. Harper, J.R.E. Davis, M.R.H. White, A.J. Millar, and D.A. Rand. Reconstruction of transcriptional dynamics from gene reporter data using differential equations. *Bioinformatics*, 24(24):2901, 2008.

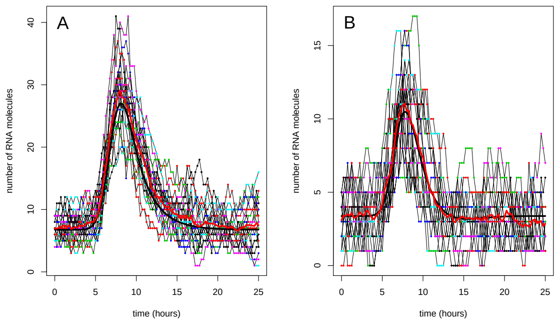

Figure 2: Timeseries of mRNA generated using Gillespie algorithm for models of single gene expression without autoregulation **A** and with autoregulation **B**. Parameters used for simulation and estimates inferred from the timeseries are presented in Tables 1A and 1B in the main text. In each panel 20 timeseries are presented. Deterministic trajectory and average trajectory are plotted in bold black and red respectively. Corresponding protein trajectories used for inference are presented in the main text.
